# A new insight for stem cell therapy: apoptotic stem cells as a key player

**DOI:** 10.1038/s41392-022-01066-z

**Published:** 2022-08-28

**Authors:** Yuhe Huang, Ziyi Bai, Kang Zhang

**Affiliations:** 1 Laboratory of Aging Research and Cancer Drug Target, State Key Laboratory of Biotherapy, National Clinical Research Center for Geriatrics, West China Hospital, Sichuan University, No. 17, Block 3, Southern Renmin Road, Chengdu, Sichuan 610041 PR China; 2 Zhuhai International Eye Center, Zhuhai People’s Hospital and The First Affiliated Hospital of Faculty of Medicine, Macau University of Science and Technology, Zhuhai, China

**Keywords:** Mesenchymal stem cells, Stem-cell research

Recently, two studies on therapeutic effects of stem cell therapy were published in S*ignal Transduction and Target Therapy*^1^ and *Nature Communications*.^2^ These studies have shed the light on a unique aspect of stem cell therapy which involves a previously unrecognized key player, namely apoptotic cells. These two publications have elucidated how the apoptotic cells exert immunomodulatory effects in stem cell therapy in several animal models.

Mesenchymal stem/stromal cells (MSCs) have a great potential for clinical applications, primarily in tissue repair and regeneration. The existence of a population of bone marrow stromal cells in the bone marrow was first identified by Friedenstein et al. in the late 1960s. In 1991, Caplan identified these cells as stem cells and named them mesenchymal stem cells.^3^ Mesenchymal stromal cells (MSCs) were first used as cellular drugs to treat human diseases by Hillard Lazarus in 1995. Since then, many studies have shown that MSCs transplantation can enable the treatment of a variety of diseases characterized by cell damage or loss, such as traumatic brain injury, Parkinson’s disease, type 1 diabetes, and liver lesions. In addition, MSCs are also an excellent vector for anti-cancer gene transfer.^4^ Due to their unique ability to migrate directionally into damaged tissues and modulate the immune response according to specific context situations, MSCs have many clinical applications such as treating graft-versus-host disease (GvHD) and autoimmune diseases.^5^ MSCs can regulate the adaptive and innate immunity by interacting with immune cells (T cells, B cells, macrophages, etc.). In most MSC-related immunological studies, MSCs were given by an intravenous injection, and they exhibit a short half-life in the body and are mostly undetectable within a few hours after an injection. Previous investigations have reported that stem cells undergo apoptosis following an injection and apoptotic stem cells may retain immunomodulatory properties.^6^

In July 2021, He et al. published an article on S*ignal Transduction and Target Therapy*, demonstrating that treatment with apoptotic MSCs alone had similar therapeutic effects to live MSCs. To investigate whether dead MSCs (DMSCs) exhibit similar immunomodulatory effects to that by live MSCs, MSCs or DMSCs with various concentrations were intravenously infused into mice with an acute live injury caused by a concanavalin A (ConA) treatment. Administration of DMSCs alone displayed the same hepatoprotective functional properties as live MSCs in multiple aspects, including improvement in hepatic lobule structure, area reduction of necrotic and apoptotic hepatocytes, and decrease of abnormal values in serum alanine aminotransferase (ALT) and aspartate aminotransferase (AST). Moreover, in mice treated with DMSCs, pro-inflammatory cytokines (IL-6, IFN-γ, and TNF-α) were reduced, while anti-inflammatory cytokines (IL-10) and hepatocyte growth factor (HGF) were elevated. Similar to MSCs, treatment with DMSCs alone increased the survival rate of mice with acute liver injury. Additionally, the authors confirmed that immunomodulatory effects of DMSCs were adaptable to a variety of insults including carbon tetrachloride (CCl4)-induced acute liver injury, LPS-induced lung injury, and spinal cord injury, implying that anti-inflammatory effects of apoptotic MSCs might be broad-spectrum.

To determine the fate of MSCs in vivo following injection, MSCs labeled with green fluorescent protein (GFP) were injected into mice. Consistent with previous research, most transplanted MSCs undergo apoptosis within 12 h in vivo. It is intriguing to identify that phosphatidylserine (PS), a representative marker of apoptosis, was significantly elevated following MSC and DMSC injection. These findings motivated authors to investigate whether the released PS plays a significant role in the protective effects of DMSCs. As expected, administration of PS liposomes (PSLs) had similar therapeutic effects to MSCs and DMSCs in mice models of acute liver and lung injuries.

**Fig. 1:**
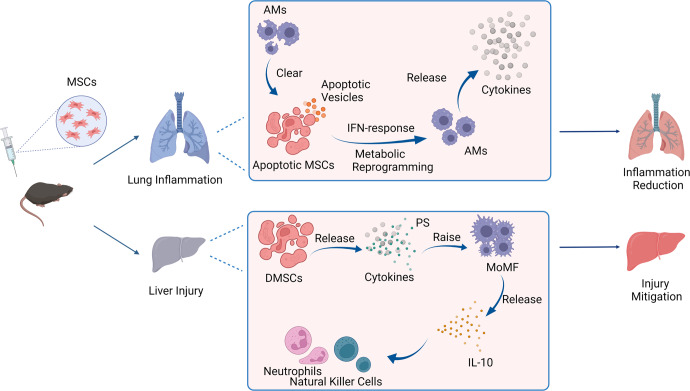
Apoptosis is necessary for immunomodulatory functions of MSCs therapy. After MSC injection, apoptotic MSCs were found in the lung. Alveolar macrophages (AMs) engulf the apoptotic cells and release cytokines to exert immune effects, reducing inflammation. In the liver, dead MSCs (DMSCs) release cytokines, of which, the released phosphatidylserine (PS) recruits monocyte-derived macrophages (MoMF) to release IL-10, acting on natural killer cells and neutrophils to exert an immune effect. Created with BioRender.com

Tyro3, Axl, and Mer tyrosine kinase (TAM) families are the main receptors for PS recognition. To explore the role of TAM receptors in immunomodulatory ability of MSCs, DMSCs, and PS, this research utilized LDC1267, a TAM receptor-specific inhibitor. LDC1267 pretreatment blocked hepatoprotective functions of MSCs, DMSCs, and PS. The authors further confirmed that Mer tyrosine kinase (MerTK), rather than Tyro3 or AXL, is the imperative receptor for these immunomodulatory effects. MSCs and DMSCs lost their hepatoprotective functions in MerTK-knockout mice. As a result, He et al. proposed that MSCs/DMSCs reshape liver immune-microenvironment via PS release. Activated natural killer (NK) cells and neutrophils were remarkably decreased after MSCs, DMSCs, and PS treatment. Additionally, DMSCs and PSLs promote macrophage polarization into M2 phenotypes. Further, He et al. discovered that a group of IL-10-producing monocyte-derived macrophages (MoMF) may play a critical function in immunosuppressive effects of MSCs and that chemokine receptor C–C motif chemokine receptor 2 (CCR2) is partly responsible for their recruitment (Fig. 1).^1^

In accordance with above results, a recent study published in *Nature Communications* revealed that mesenchymal stromal cells undergo in vivo apoptosis to exert immunomodulatory effects. MSCs lodged in the lung and rapidly underwent apoptosis following an intravenous administration. The authors demonstrated that the clearance of MSCs from lung is unrelated to immune cell cytotoxicity and that allorecognition is not the main mechanism underlying this process. However, MSCs are taken up by phagocytic cells in the lungs, including neutrophils, monocytes, and alveolar macrophages (AMs). Interestingly, this study demonstrated that injecting apoptotic MSCs induced by staurosporine (STS) or a combination of BH3-mimetic drugs (targeted BCL-2, BCL-XL, and MCL-1) had similar immunosuppressive capacity to that of viable MSCs in multiple inflammatory models, such as asthma and airway hyperresponsiveness. Remarkably, deficiency of apoptosis promoting factors BAK and BAX prevents MSCs from undergoing apoptosis and alleviates their immunomodulatory effects. Finally, the authors underlined the critical role of AMs in MSC clearance, noting that apoptotic MSCs undergo efferocytosis by AMs, which targets metabolic and inflammatory pathways (Fig. 1). This research reveals that while apoptotic MSCs may not have direct immunosuppressive effects, they could release extracellular vesicles (including apoptotic vesicles) to regulate an immune response. For instance, apoptotic MSCs may recruit immunomodulatory macrophages, regulatory T cells, or B cells to produce anti-inflammatory cytokines and modulate numerous aspects of immune function.^2^

Stem cell therapy encounters many challenges, such as improper extraction and preservation, and ethical issues raised by stem cell transplantation. In addition, there is an urgent need to improve the activity of stem cells, and the risk of promoting tumor growth still exists. Mesenchymal stem cells are widely available with a good safety profile, pose few ethical concerns, can be expanded in vitro at a large scale, and can be commercialized toward clinical treatment in the future.^7^ However, MSCs rapidly disappear following transplantation and function via paracrine and post-apoptotic products. Based on the above studies, it is worth exploring to clarify which components of the apoptotic stem cells themselves, as well as the released substances, play a major role in the treatment of diseases. Products of Exosomes and post-apoptotic produced from MSCs and DMSCs exhibit therapeutic properties comparable to those of MSCs, and they are durable, easy to preserve, and less prone to risks of immune rejection and tumor formation. Therefore, the option to use exosomes, dead cells, or even cellular debris as a therapeutic tool may be a viable one. In addition, converting a single component into a therapeutic agent may be a more effective and promising alternative therapy that has the potential of transforming a conventional MSC therapy into a cell-free therapy. However, whether injecting apoptotic cells into the body to repair damaged tissues will cause other adverse consequences on the immune system or other organs of the body remains an open question. The above studies provide a new exciting avenue for stem cell therapy in repair and regeneration, and in the combination of drug delivery and gene editing technologies, may broaden the applications of MSC therapies.
